# Clinical implications of the tumor microenvironment using multiplexed immunohistochemistry in patients with advanced or metastatic renal cell carcinoma treated with nivolumab plus ipilimumab

**DOI:** 10.3389/fonc.2022.969569

**Published:** 2022-09-27

**Authors:** Jwa Hoon Kim, Gi Hwan Kim, Yeon-Mi Ryu, Sang-Yeob Kim, Hyung-Don Kim, Shin Kyo Yoon, Yong Mee Cho, Jae Lyun Lee

**Affiliations:** ^1^ Department of Oncology, Asan Medical Center, University of Ulsan College of Medicine, Seoul, South Korea; ^2^ Division of Oncology, Department of Internal Medicine, Korea University Anam Hospital, Korea University College of Medicine, Seoul, South Korea; ^3^ Department of Pathology, Asan Medical Center, University of Ulsan College of Medicine, Seoul, South Korea; ^4^ Department of Convergence Medicine, University of Ulsan College of Medicine, Asan Medical Center, Seoul, South Korea

**Keywords:** renal cell carcinoma, tumor microenvironment, immune checkpoint inhibitors, response, survival

## Abstract

**Purpose:**

Immune checkpoint inhibitors (ICIs) such as nivolumab and ipilimumab (N/I) are important treatment options for advanced renal cell carcinoma (RCC). The tumor microenvironment (TME) in these ICI-treated patients is largely unknown.

**Methods:**

Twenty-four patients treated with N/I between July 2015 and June 2020 were analyzed. Multiplexed immunohistochemistry (mIHC) was conducted to define the TME, including various T cell subsets, B cells, macrophages, and dendritic cells.

**Results:**

The median age of the study patients was 61 years (range, 39–80) and 75.0% of these cases were men. The objective response rate with N/I was 50.0%. The densities of the CD8+ cytotoxic T cells (*P*=0.005), specifically CD137+ CD8+ T cells (*P*=0.017), Foxp3- CD4+ helper T cells (*P*=0.003), Foxp3+ CD4+ regulatory T cells (*P*=0.045), CD68+ CD206- M1 macrophages (*P*=0.008), and CD68+ CD206+ M2 macrophages (*P*=0.021) were significantly higher in the treatment responders. At a median follow-up duration of 24.7 months, the median progression-free survival (PFS) was 11.6 months. The high densities (≥median) of Foxp3- CD4+ helper T cells (*P*=0.016) and CD68+ CD206- M1 macrophages (*P*=0.008) were significantly associated with better PFS, and the density of CD137+ CD8+ cytotoxic T cells (*P*=0.079) was marginally associated with better PFS. After multivariate analysis, the higher density of Foxp3- CD4+ helper T cells was independently associated with better PFS (hazard ratio 0.19; *P*=0.016).

**Conclusion:**

The properties and clinical implications of the TME properties in RCC indicate that Foxp3- CD4+ helper T cells, M1 macrophages, and CD137+ CD8+ T cells are potential predictive biomarkers and treatment targets.

## Introduction

The prognosis of advanced renal cell carcinoma (RCC) has considerably improved in recent decades due to the introduction of immune checkpoint inhibitors (ICIs), which block programmed death (ligand) 1 (PD-L1) and cytotoxic T lymphocyte antigen-4 (CTLA4) and combinations of ICI plus vascular endothelial growth factor receptor tyrosine kinase inhibitor. Following the phase III CheckMate-214 trial, a first-line therapy with nivolumab (an anti-PD-1 inhibitor) plus ipilimumab (an anti-CTLA4 inhibitor), compared to sunitinib alone, was found to improve the objective response rate (ORR) (42% vs. 27%, *P*<0.001) and overall survival (OS) (hazard ratio [HR] 0.63, *P*<0.001) in intermediate- and poor-risk patients (1). The long-term follow-up analysis of these trial subjects also demonstrated durable efficacy benefits with nivolumab plus ipilimumab compared with sunitinib (2, 3). However, only a limited number of patients benefit from ICIs. The ORR was 42% with nivolumab plus ipilimumab versus 27% with sunitinib (*P*<0.001). Approximately 20% (83/425) of the intermediate- and poor-risk patients from the CheckMate-214 trial (1) experienced initial disease progression and had relatively short progression-free survival (PFS).

There are currently no validated biomarkers for predicting the ICI treatment response. The predictive and prognostic significance of PD-L1 expression, genomic mutations, the tumor mutation burden, and gene expression patterns have previously been explored in ICI-treated patients (4–7). The peripheral blood markers such as absolute neutrophil, lymphocyte, monocyte, eosinophil, and immune cell counts have been also investigated for the prediction of response to ICI treatment (8–10). However, understanding the determinants of these treatment responses is challenging. Given that the tumor microenvironment (TME) can influence the response to ICIs, an investigation of its heterogeneous characteristics is necessary to predict this response, and a better understanding of the underlying immunity in the patients could suggest novel strategies to further improve clinical outcomes (11, 12).

Among various immune subsets in TME, T cell subsets such as cytotoxic CD8+ T cells, helper CD4+ T cells, and regulatory CD4+ T cells are recognized as key components in the anti-tumor immune response (13–15). CD8+ T cells are activated through the CD137 signaling, thereby enhancing T cell survival and promoting their effector function (16). Macrophages, dendritic cells, and B cells also participate in antigen presentation, inflammation, and anti-tumor activity (17). Previous studies have examined the prognostic value of various immune subsets using conventional immunohistochemistry (IHC) in various cancer (18, 19). However, conventional IHC has limitations in that it is impossible to stain multiple markers at once on the same specimen slide to evaluate immune subsets and cannot evaluate immune cell counts. The multiplexed IHC (mIHC) is the quantitative multispectral imaging method that can discriminate immune subsets based on the expression of multiple markers. This novel method has been validated to reflect conventional IHC-based immune cell evaluation and is increasingly used to assess the immune profiles of the TME (20, 21).

In our present study, we performed mIHC to investigate the features of TME in patients with advanced RCC receiving nivolumab plus ipilimumab and evaluated the prognostic implications for the prediction of a treatment response.

## Materials and methods

### Patients

A total of 24 patients with advanced or metastatic RCC were treated with nivolumab plus ipilimumab as first-line therapy at Asan Medical Center, Seoul, Republic of Korea, between July 2015 and June 2020. mIHC was retrospectively performed to investigate the characteristics of TME in these patients. This retrospective study was approved by the Institutional Review Board of Asan Medical Center (study number: 2019-1712), and it was conducted in accordance with the Declaration of Helsinki and Good Clinical Practice.

Patients with International Metastatic RCC Database (IMDC) (22) at intermediate- or poor-risk received nivolumab (3 mg/kg) and ipilimumab (1 mg/kg) intravenously as a first-line therapy every 3 weeks in four doses, followed by nivolumab (3 mg/kg) every 2 weeks. The tumor response was assessed using computed tomography every 6 to 9 weeks for the first year and then every 9 to 12 weeks thereafter until disease progression or discontinuation of ICI treatment, based on the response evaluation criteria in solid tumors (RECIST) criteria v1.1 (23).

### Multiplexed immunohistochemistry

Optimized fluorescent mIHC was performed by tyramide signal amplification (TSA) using a Leica Bond Rx™ Automated Stainer (Leica Biosystems, Newcastle, UK). Cells were stained with antibodies against CD20 (ab9475; Abcam, Cambridge, UK), CD4 (ab133616; Abcam), CD103 (ab129202; Abcam), Foxp3 (ab20034; Abcam), CD137 (ab197942; Abcam), CD8 (MCA1817; Bio-Rad, Hercules, CA, USA), CD206 (NBP1-90020; Novus Biologicals, Littleton, CO, USA), CD68 (ab 192847; Abcam), CD11c (ab52632; Abcam), MHCII (ab 7856; Abcam), and PD-L1 (13684S; Cell Signaling Technology, Danvers, MA, USA). The fluorescence signals were captured with the following fluorophores: Opal 480, Opal 520, Opal 570, Opal 620, Opal 690, and Opal 780. Multiplex-stained slides were obtained using the Vectra^®^ Polaris Quantitative Pathology Imaging System (PerkinElmer, Boston, MA, USA). The images were analyzed using inForm 2.4.4 image analysis software (PerkinElmer) and Spotfire™ software (TIBCO Software Inc., Palo Alto, CA, USA).

**Figure 1:**
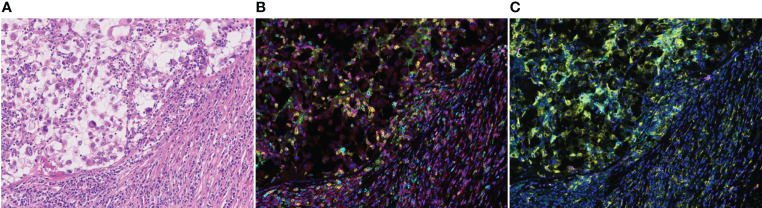
Representative examples of multiplexed immunohistochemical staining of advanced renal cell carcinoma tissue sections. **(A)** hematoxylin and eosin staining. **(B)** CD20, CD4, CD103, Foxp3, CD137, and CD8. **(C)** CD206, CD68, CD11c, MHCII, and PDL1. Original magnification, x 200.

Regions of interest (ROIs) representing each tissue specimen were carefully chosen by pathologists, based on hematoxylin and eosin slides, and approximately 7–11 ROIs were thereby selected for each tissue specimen. We also subdivided the tumor into center, margin, and stroma regions in the available tissues from surgical specimens. The immune cell activity and its clinical value may be different according to the spatial distribution. Representative images are shown in **Figure 1**, and the implications for each marker are explained in **Table S1**. CD8+ was used for indicating cytotoxic T cells; CD103+ CD8+ for tissue-resident T cells and CD137+ CD8+ or CD137+ CD4+ for costimulatory 4-1BB-expressing T cells, both used as activated T cells; Foxp3- CD4+ for helper T cells; Foxp3+ CD4+ for regulatory T cells; CD20+ for B cells; CD206- CD68+ for M1-polarized macrophages; CD206+ CD68+ for M2-polarized macrophages; CD11c+ MHC class II+ for antigen-presenting dendritic cells; and PD-L1+ for immune regulatory molecules. Cell densities are measured as the mean/mm^2^ for each cell population.

### Statistical analyses

Categorical and quantitative data were compared using the chi-square test or Fisher’s exact test, and Mann–Whitney U tests. The mean levels of the markers among the three groups were compared using analysis of variance (ANOVA). Multiple comparison tests were not performed. The PFS was calculated from the date of ICI initiation to the date of disease progression or death from any cause, whichever occurred first. The OS was calculated from the date of ICI initiation to the date of death from any cause. Survival was estimated using the Kaplan–Meier method, and the log-rank test was used to compare the differences between the curves. A two-sided *P*-value <0.05 was considered significant, and all statistical analyses were performed using the statistical package for the social sciences (SPSS) 25.0 software package (IBM SPSS Statistics, Chicago, IL, USA).

## Results

### Patient characteristics

A total of 24 patients underwent mIHC analysis in this study. The baseline patient characteristics are summarized in **Table 1**. The median patient age was 61 years (range, 39–80 years), and 75.0% were men. The available tissues were obtained prior to nivolumab plus ipilimumab treatment. Tissues were obtained from surgery (n=16) or biopsy (n=8).

**Table 1:** Baseline characteristics of the study patients and clinical outcomes with nivolumab plus ipilimumab.

	Total patients (n=24, %)
Median age, years (range)	61 (39–80)
Sex	
male	18 (75.0)
female	6 (25.0)
IMDC risk group	
Intermediate	14 (58.3)
Poor	10 (41.7)
Histology type	
Clear cell*	23 (95.8)
Presence of sarcomatoid component	8 (33.3)
Site of metastasis	
Lymph node	10 (41.7)
Lung	19 (79.2)
Liver	3 (12.5)
Bone	10 (41.7)
Previous nephrectomy	17 (70.8)
**Response and survival with nivolumab plus ipilimumab**	
Complete response	3 (12.5)
Partial response	9 (37.5)
Stable disease	5 (20.8)
Progressive disease	7 (29.2)
Objective response rate	12 (50.0)
Disease control rate	17 (70.8)
Median progression-free survival	11.6 (95% CI 5.2–17.9) months
Median overall survival	Not reached

IMDC; International Metastatic RCC Database Consortium, CI; confidence interval

*One patient had a sarcomatoid renal cell carcinoma.

**Table 1** summarizes the efficacy of the ICI treatments. The ORR and disease control rate (DCR) were 50.0% and 70.8%, respectively. At a median follow-up duration of 24.7 months (95% confidence interval [CI], 21.5–28.0), 14 patients (58.3%) experienced disease progression and the median PFS was 11.6 (95% CI, 5.2–17.9) months. The median OS was not reached because only five (20.8%) patients had died at the time of the analysis.

### Association of tumor microenvironment immune cells with responses to nivolumab plus ipilimumab

The densities of the T cell subsets, B cells, macrophages, dendritic cells, and PD-L1-expressing immune cells were compared between responders (complete response [CR] + partial response [PR]) and non-responders. The density of immune cells in the TME of the advanced RCC lesions is listed according to the response in **Table 2**. The density of CD8+ cytotoxic T cells (*P*=0.005), Foxp3- CD4+ helper T cells (*P*=0.003), and Foxp3+ CD4+ regulatory T cells (*P*=0.045) was significantly higher in responders than in non-responders. Specifically, CD137+ CD8+ T cells (*P*=0.017) was highly infiltrated in the responders. A high infiltration of CD68+ CD206- M1 macrophages or CD68+ CD206+ M2 macrophages was significantly associated with achieving a response to nivolumab plus ipilimumab (*P*=0.008 and *P*=0.021). Otherwise, there were no significant differences in the density of CD11c+ MHC class II+ dendritic cells or PD-L1-expressing immune cells between the responders and non-responders.

**Table 2:** Immune cell infiltration densities between the treatment responders and non-responders.

	Nivolumab plus ipilimumab (n=24, %)		
	Responders (n=12),median (IQR 25%-75%)	Non-responders (n=12),median (IQR 25%-75%)	*P*-value
CD8+ cytotoxic T cells	394.2 (157.7-670.4)	98.1 (50.4-279.3)	0.005
CD103+ CD8+ tissue-resident T cells	18.2 (2.5-33.1)	8.3 (3.7-15.5)	0.148
CD137+ CD8+ T cells	5.6 (1.9-45.7)	0.9 (0.0-8.3)	0.017
Foxp3- CD4+ helper T cells	349.1 (251.2-799.6)	58.2 (25.3-147.2)	0.003
Foxp3+ CD4+ regulatory T cells	15.8 (2.3-22.6)	0.7 (0.2-3.1)	0.045
CD137+ CD4+ T cells	7.0 (2.2-120.9)	3.3 (0.0-33.5)	0.090
CD20+ B cells	21.1 (5.8-40.7)	3.3 (0.7-31.5)	0.134
CD68+ CD206- M1 macrophages	643.67 (408.95-1148.24)	126.50 (71.59-575.16)	0.008
CD68+ CD206+ M2 macrophages	3.67 (1.10-12.46)	0.63 (0.0-2.42)	0.021
CD11c+ MHC class II+ dendritic cells	0 (0-1.4)	0 (0-0)	0.557
PD-L1+ cells	770.6 (506.9-1417.7)	388.3 (92.4-1143.2)	0.223

IQR, interquartile.

Cell densities are measured as the mean/mm^2^ for each cell population.

### Association of tumor microenvironment immune cells with progression-free survival

**Figure 2:**
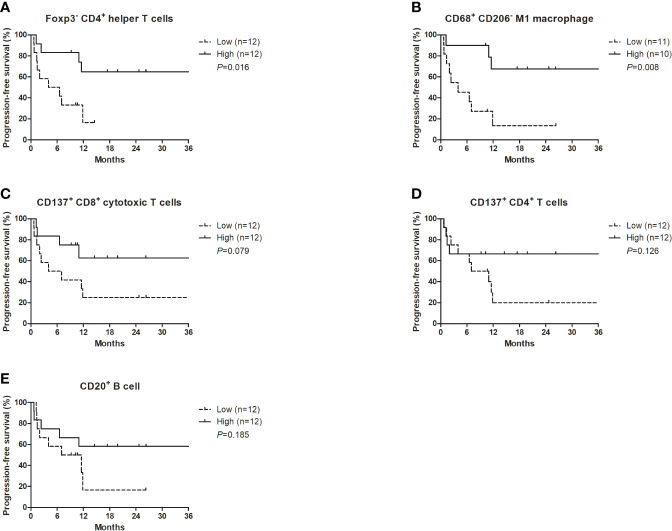
Progression-free survival with nivolumab plus ipilimumab according to the densities of certain T cell subsets, CD20^+^ B cells, and M1 macrophages at the tumor margin. **(A)** Foxp3^-^ CD4^+^ helper T cells, **(B)** CD68^+^ CD206^-^ M1 macrophages, **(C)** CD137^+^ CD8^+^ cytotoxic T cells, **(D)** CD137^+^ CD4^+^ T cells, **(E)** CD20^+^ B cells.

Each TME marker was classified into high (≥median) and low (<median) groups. The high density of Foxp3- CD4+ helper T cells (*P*=0.016) and CD68+ CD206- M1 macrophages (*P*=0.008) was significantly associated with better PFS (**Figures 2A, B**). The high density of CD137+ CD8+ cytotoxic T cells (*P*=0.079), CD137+ CD4+ cytotoxic T cells (*P*=0.126), and CD20+ B cells (*P*=0.185) was marginally associated with better PFS (**Figures 2C–E**). Multivariate analysis revealed that the higher density of Foxp3- CD4+ helper T cells was independently associated with better PFS (hazard ratio 0.19, 95% CI 0.05-0.73; *P*=0.016) (**Table 3**). There were no significant differences in the PFS according to the densities of CD11c+ MHC class II+ dendritic cells or PD-L1-expressing immune cells.

**Table 3:** Univariate and multivariate analysis for progression-free survival.

	Progression-free survival			
	Univariate analysis		Multivariate analysis*	
	HR (95% CI)	*P*-value	HR (95% CI)	*P*-value
Age (≥65 years vs. <65 years)	0.30 (0.04-2.23)	0.234		
Sex (male vs. female)	0.23 (0.08-0.69)	0.009		
IMDC (poor vs. intermediate)	2.26 (0.72-7.08)	0.162		
Presence of sarcomatoid component in histology (yes vs. no)	0.78 (0.24-2.54)	0.676		
Previous nephrectomy (yes vs. no)	0.75 (0.23-2.48)	0.642		
CD8^+^ cytotoxic T cells (high vs. low)	0.74 (0.25-2.22)	0.596		
CD103^+^ CD8^+^ tissue-resident T cells (high vs. low)	0.82 (0.27-2.47)	0.726		
CD137^+^ CD8^+^ T cells (high vs. low)	0.36 (0.11-1.18)	0.093		
CD137^+^ CD4^+^ T cells (high vs. low)	0.41 (0.13-1.34)	0.139		
FoxP3^-^ CD4^+^ helper T cells (high vs. low)	0.25 (0.07-0.83)	0.024	0.19 (0.05-0.73)	0.016
FoxP3^+^ CD4^+^ regulatory T cells (high vs. low)	0.95 (0.32-2.89)	0.934		
CD20^+^ B cells (high vs. low)	0.47 (0.15-1.47)	0.194		
CD68^+^ CD206- M1 macrophages (high vs. low)	0.19 (0.05-0.73)	0.016		
CD68^+^ CD206^+^ M2 macrophages (high vs. low)	0.40 (0.12-1.34)	0.136		
CD11c^+^ MHC class II+ dendritic cells (high vs. low)	1.13 (0.34-3.77)	0.844		
PD-L1^+^ cells (high vs. low)	0.57 (0.17-1.89)	0.359		

HR, hazard ratio; CI, confidence interval; IMDC, International Metastatic RCC Database Consortium.

*Multivariate analysis included significant factors identified by univariate analysis (P<0.1).

### Spatial distribution of tumor microenvironment immune cells

To quantify the infiltration of immune cell subsets, associated with the efficacy with nivolumab plus ipilimumab, according to their spatial distribution, the tumor regions were subdivided into a center, margin, and stroma in the available tissues (n=14). The density of FoxP3- CD4+ helper T cells, CD137+ CD8+ cytotoxic T cells, and CD137+ CD4+ T cells seemed to be numerically higher in the tumor margin than in the stroma or center (**Figure S1**).

### Association of tumor microenvironment immune cells with treatment-related adverse event to nivolumab plus ipilimumab

Treatment-related adverse event (TRAE) occurred in 16 (66.7%) (**Table S2**). The most common TRAE of any grade was rash (n=8, 33.3%) and there were grade 3 hyperglycemia (n=4, 16.7%). Common TRAE (>10%) of any grade included ALT elevation (n=7, 29.2%), AST elevation (n=5, 20.8%), anorexia (n=5, 20.8%), diarrhea (n=4, 16.7%), pruritus (n=4, 16.7%), and fatigue (n=3, 12.5%). Most of them were in grade 1. There were no significant differences in immune cell densities between patients with any grade of TRAE and those without any TRAE (**Table S3**), and patients with grade 3 hyperglyceima and those without grade ≥ 3 TRAE (**Table S4**).

## Discussion

The current study showed a significant association between the TME in RCC patients and the response and PFS to nivolumab plus ipilimumab treatment through mIHC analysis. Notably, the higher density of Foxp3- CD4+ helper T cells and CD68+ CD206- M1 macrophages was significantly associated with both the treatment response and better PFS, respectively. The density of Foxp3- CD4+ helper T cells remained a significant factor in terms of the PFS after multivariate analysis.

There is growing interest in unraveling the role of TME in identifying biomarkers but exploring its heterogeneity is a complex task in highly immune-infiltrated RCC (11, 12). A simple measurement of CD8+ T cells is unlikely to be predictive of an ICI response (11), and a defective T cell function in RCC has been reported in several studies (24–26). Emerging evidence has suggested that CD4+ T cells may also play a critical role in immune responses. Foxp3- CD4+ helper T cells have been shown to promote the priming of tumor-specific CD8+ T cells and help elicit durable T cell responses by interacting with dendritic cells in an MHCII-dependent manner (14). CD68+ CD206- M1 macrophages participate in antigen presentation, inflammation, and anti-tumor activity (27). We found also in our current analyses that CD137+ CD8+ T cells, as a population of activated T lymphocytes, had a significantly higher level of infiltration in the responders compared with the non-responders, and that this higher density was marginally associated with better PFS. It is well known that signaling through CD137 induces the activation of CD8+ T cells, thereby enhancing T cell survival, promoting their effector function, and favoring memory differentiation (28). Regarding the Foxp3+ CD4+ regulatory T cells known to have opposing roles in antitumor immunity (14), we found in our present analyses that the density of Foxp3+ CD4+ regulatory T cells was inversely higher in responders than in non-responders. This may be explained by the fact that the antitumor activity of anti-CTLA4 inhibitors is dependent on the depletion of CTLA4-expressing regulatory T cells in the TME through antibody-dependent cellular cytotoxicity (29). Hence, patients with a higher density of Foxp3+ CD4+ regulatory T cells can be more susceptible to anti-CTLA4 inhibitors. It has been reported in this regard that a higher Foxp3+ CD4+ regulatory T cell level at baseline is significantly associated with favorable outcomes with ipilimumab therapy in patients with melanoma (30).

Exploratory biomarker studies (4–7) using pivotal trials, including CheckMate-214 (1) and CheckMate-025 (31, 32), have been conducted to predict ICI treatment responses. In the CheckMate-214 trial, PD-L1 IHC, whole exome sequencing and RNA sequencing were performed to evaluate PD-L1 positivity, tumor mutation burden, indel burden, human leucine antigen class I zygosity, the PBRM1 mutation status, and gene signature scores (4). Although the tumor mutation burden and genomic instability can serve as robust predictors of an ICI response in various cancers, these expected factors, as well as PD-L1 positivity, were not found previously to be associated with the clinical benefits of a nivolumab plus ipilimumab combination (4). Besides the PD-1/PD-L1 axis and CTLA-4 for these checkpoint inhibitors, there are several other checkpoints such as PD-L2, T cell immunoglobulin and mucin domain containing 3 (TIM3), and lymphocyte activating 3 (LAG3), which may be associated with immune response (33–35). In the CheckMate-025, -010, and -009 trials, the tumor mutation burden and CD8+ T cell infiltration level were not predictive of second-line nivolumab monotherapy in patients previously treated with tyrosine kinase inhibitor (5–7). However, these predictive values may vary depend on treatment settings and types of ICIs. In this study, the combination of nivolumab with ipilimumab was administered as first-line, and different from nivolumab monotherapy, limited the determination of its predictive values. Unlike previous studies, we here directly examined various immune cells in RCC tissue samples that are the major players in the TME associated with antitumor activity. Moreover, our mIHC approach enhanced the quality of the TME analysis, considering that the difference between certain T cell subsets is not detectable by conventional IHC.

It has been proposed that with the investigation of specific TME components and their recognized impact on the treatment responses, combination strategies that target distinct immune cell subsets may help overcome treatment resistance (11). Repolarizing macrophages toward an M1 phenotype could promote an immune response and engender synergistic effects with ICIs. Inhibitors of PI3Kγ or mTOR as well as agonists of CD40, TLR4, -7, -8, or -9 can repolarize macrophages towards a proinflammatory phenotype promoting tumor suppression in preclinical studies (36). Considering that the indolamine 2,3 dioxygenase 1 (IDO1) overexpressed by M2 macrophages depletes the essential metabolite tryptophan, which hampers T cell proliferation (37), the combination of epacadostat (IDO1 inhibitor) and pembrolizumab has showed promising results, with an ORR of 47% in 19 patients with advanced RCC previously treated with antiangiogenic agents, irrespective of their risk groups (38). The combination of epacadostat and ipilimumab has also shown a promising ORR of 23% in immunotherapy-naïve melanoma patients (39). The efficacy of the combination of epacadostat with ICIs needs to be further investigated, focusing only on intermediate- or high-risk RCC patients. Moreover, along with the prognostic value of CD137, the efficacy and safety of CD137 agonists alone or in combination with ICIs have been investigated in several studies (40–42). Novel therapeutic strategies targeting the upregulation of CD137 expression or enhancement of CD137 signaling for synergistic effects with ICIs need to be further studied in advanced RCC.

Despite our subgroup analysis with further small samples, significant numbers of immune cells had a trend of higher infiltration in the tumor margin than in the tumor center and stroma. The clinical value of the spatial distribution of immune cells has been reported for other cancer types. The density of Foxp3- CD4+ helper T cells in the tumor margin rather than the tumor center and stroma has previously shown the best capacity for predicting the treatment response in biliary tract cancer patients, and the tumor margin may be the main site of the immune response in these cases (43).

The present study had some limitations of note. First, only a small number of patients treated with nivolumab plus ipilimumab were included. This regimen was of limited use because it is not covered yet by the National Health Insurance Service of Korea when this study was designed. Further, larger-scale studies are needed to confirm the value of significant TME biomarkers. Second, only approximately one in five patients in our cohort died at the time of the analysis and OS data could not therefore be analyzed. Long-term follow-up is necessary because PFS cannot always guarantee a long-term response. Third, TME analysis using mIHC may not represent the entire tissue specimen because it is limited to ROIs. There are particular concerns in this regard when using biopsy specimens rather than surgical specimens. It may be necessary to investigate a wider area of tumor tissues to properly assess any possible clinical applicability of these findings, as well as to validate TME biomarkers associated with an ICI treatment response.

In conclusion, several immune cells in the TME are fully associated with the response to ICIs, particularly Foxp3- CD4+ helper T cells and M1 macrophages. These are new predictive biomarkers and possible future therapeutic targets that could help to further improve survival.

## Data availability statement

The raw data supporting the conclusions of this article will be made available by the authors, without undue reservation.

## Ethics statement

The studies involving human participants were reviewed and approved by Institutional Review Board of Asan Medical Center (study number: 2019-1712). The ethics committee waived the requirement of written informed consent for participation.

## Author contributions

Study concepts: JL. Study design: JK and JL. Data acquisition: JK, GK, Y-MR, S-YK, H-DK, SY, YC, and JL, Quality control of data and algorithms: JK, GK, Y-MR, and YC. Data analysis and interpretation: JK, GK, and Y-MR. Statistical analysis: JK and GK, Manuscript preparation: JK and GK. Manuscript editing: JK, GK, and JL, Manuscript review: JK, GK, Y-MR, S-YK, H-DK, SY, YC, and JL. All authors contributed to the article and approved the submitted version.

## Conflict of interest

The authors declare that the research was conducted in the absence of any commercial or financial relationships that could be construed as a potential conflict of interest.

## Publisher’s note

All claims expressed in this article are solely those of the authors and do not necessarily represent those of their affiliated organizations, or those of the publisher, the editors and the reviewers. Any product that may be evaluated in this article, or claim that may be made by its manufacturer, is not guaranteed or endorsed by the publisher.

## Supplementary material

Supplementary Figure 1 Quantification of the infiltration level by certain T cell subsets, CD20^+^ B cells, and M1 macrophages, according to the spatial distribution in each available dataset.

Supplementary Table 1 Implications for each markers of multiplexed immunohistochemistry.

Supplementary Table 2 Treatment-related adverse event.

Supplementary Table 3 Immune cell infiltration densities between patients with any grade of TRAE and those without any TRAE.

Supplementary Table 4 Immune cell infiltration densities between patients with grade 3 hyperglycemia and those without grade ≥ 3 TRAE.
