# Supplementary figures and images for: Clinical implications of the tumor microenvironment using multiplexed immunohistochemistry in patients with advanced or metastatic renal cell carcinoma treated with nivolumab plus ipilimumab
Source: Front Oncol. 2022 Sep 27;12:969569. doi: 10.3389/fonc.2022.969569 (PMC9552830; doi:10.3389/fonc.2022.969569)

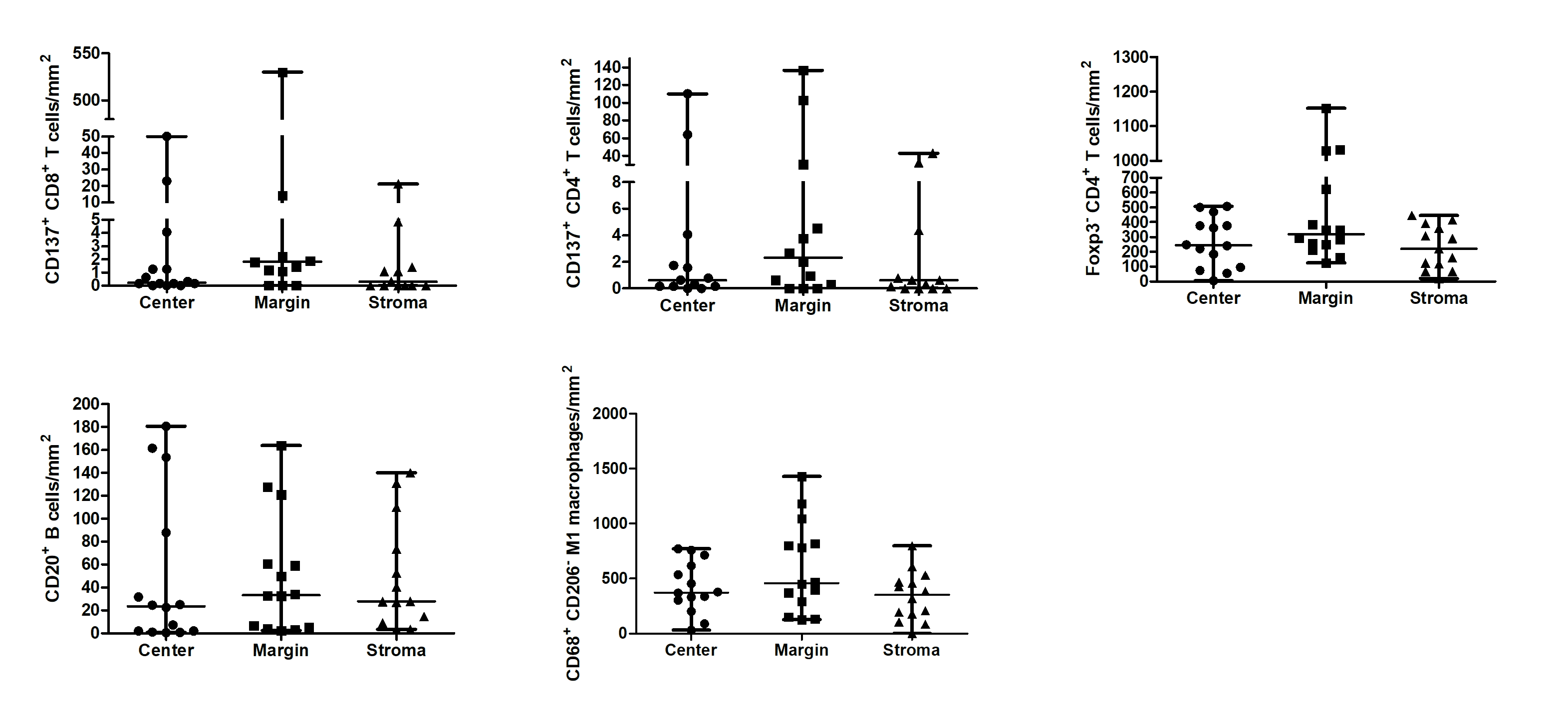

Supplement: Supplementary Figure 1 — Quantification of the infiltration level by certain T cell subsets, CD20+ B cells, and M1 macrophages, according to the spatial distribution in each available dataset. [file Image_1.tif]
