# Supplementary material for: Clinical implications of the tumor microenvironment using multiplexed immunohistochemistry in patients with advanced or metastatic renal cell carcinoma treated with nivolumab plus ipilimumab
Source: Front Oncol. 2022 Sep 27;12:969569. doi: 10.3389/fonc.2022.969569 (PMC9552830; doi:10.3389/fonc.2022.969569)
Supplement: Supplementary Table 1 — Implications for each markers of multiplexed immunohistochemistry. [file Table_1.docx]

Supplementary Tables

Table S1. Implications for each markers of multiplexed immunohistochemistry.

| Antibody used in panel 1 | Implication |
| --- | --- |
| CD8 | Found on the surface of cytotoxic T cells |
| CD4 | Found on the surface of helper T cells or regulatory T cells |
| Foxp3 | Functions as a master regulator of the regulatory pathway in the development and function of regulatory T cells |
| CD103 | Found on the surface of intraepithelial lymphocyte T cells |
| CD137 | Costimulatory activity for activated T cells and found on the surface of CD8+ and CD4+ lineages |
| CD20 | Found at all stages of B cell development and functions to promote an increased B cell immune response |
| Antibody used in panel 2 |  |
| CD68 | Found on the surface of all macrophages, including the M1 and M2 phenotypes |
| CD206 | Found on the surface of macrophages and distinguishes the M1 and M2 phenotypes |
| CD11c | Found on the surface of dendritic cells |
| MHCII | Found on the surface of professional antigen-presenting cells |
| PD-L1 | Found on the surface of tumor cells or immune cells |

Table S2. Treatment-related adverse event.

|  | Any grade | Grade 3 or 4 |
| --- | --- | --- |
| Neutropenia | 0 (0) | 0 (0) |
| Anemia | 0 (0) | 0 (0) |
| Thrombocytopenia | 0 (0) | 0 (0) |
| AST elevation | 5 (20.8) | 0 (0) |
| ALT elevation | 7 (29.2) | 0 (0) |
| Amylase elevation | 1 (4.2) | 0 (0) |
| Lipase elevation | 2 (8.3) | 0 (0) |
| Hyperglycemia | 4 (16.7) | 4 (16.7) |
| Hypothyroidism | 0 (0) | 0 (0) |
| Fatigue | 3 (12.5) | 0 (0) |
| Myalgia | 1 (4.2) | 0 (0) |
| Anorexia | 5 (20.8) | 0 (0) |
| Nausea | 0 (0) | 0 (0) |
| Diarrhea | 4 (16.7) | 0 (0) |
| Pruritis | 4 (16.7) | 0 (0) |
| Rash | 8 (33.3) | 0 (0) |

Table S3. Immune cell infiltration densities between patients with any grade of TRAE and those without any TRAE.

|  | Nivolumab plus ipilimumab (n=24, %) | | |
| --- | --- | --- | --- |
|  | Any grade of TRAEs (n=16),  median (IQR 25%-75%) | No TRAEs (n=8),  median (IQR 25%-75%) | P-value |
| CD8+ cytotoxic T cells | 131.3 (53.8-400.0) | 311.4 (145.5-516.9) | 0.452 |
| CD103+ CD8+ tissue-resident T cells | 8.8 (2.3-27.8) | 13.7 (4.3-20.7) | 0.976 |
| CD137+ CD8+ T cells | 1.8 (0-10.3) | 2.0 (1.2-28.2) | 0.653 |
| Foxp3- CD4+ helper T cells | 149.7 (40.6-443.3) | 205.9 (67.3-298.8) | 0.928 |
| Foxp3+ CD4+ regulatory T cells | 1.4 (0.4-12.4) | 8.5 (0.8-20.6) | 0.681 |
| CD137+ CD4+ T cells | 1 (0.2-50.1) | 6.0 (4.3-66.7) | 0.291 |
| CD20+ B cells | 6.5 (1.8-55.2) | 7.5 (3.6-18.3) | 0636 |
| CD68+ CD206- M1 macrophages | 380 (292.6-721.7) | 433.1 (105.0-614.6) | 0.585 |
| CD68+ CD206+ M2 macrophages | 2 (0.7-2.9) | 0.6 (0.5-5.4) | 0.535 |
| CD11c+ MHC class II+ dendritic cells | 0 (0-0.5) | 0 (0-0.5) | 0.689 |
| PD-L1+ cells | 495.2 (210.2-1417.2) | 683.9 (462.2-1026.8) | 0.913 |

Table S4. Immune cell infiltration densities between patients with grade 3 hyperglycemia and those without grade $\geq$3 TRAE.

|  | Nivolumab plus ipilimumab (n=24, %) | | |
| --- | --- | --- | --- |
|  | Grade 3 hyperglycemia (n=4),  median (IQR 25%-75%) | No grade $\geq$3 of TRAEs (n=20),  median (IQR 25%-75%) | P-value |
| CD8+ cytotoxic T cells | 114.8 (54.9-156.6) | 283.3 (98.0-458.1) | 0.406 |
| CD103+ CD8+ tissue-resident T cells | 14.7 (2.7-21.7) | 13.3 (3.6-24.7) | 0.953 |
| CD137+ CD8+ T cells | 2.8 (1.8-9.0) | 1.9 (0.3-33.3) | 1.000 |
| Foxp3- CD4+ helper T cells | 150.0 (54.8-345) | 210.8 (53.5-368.3) | 0.783 |
| Foxp3+ CD4+ regulatory T cells | 10.5 (2.5-21.3) | 1.3 (0.4-17.6) | 0.557 |
| CD137+ CD4+ T cells | 2.7 (0.7-9.4) | 4.7 (0.6-87.7) | 0.679 |
| CD20+ B cells | 19.7 (6.5-33.1) | 5.3 (1.8-54.3) | 0.557 |
| CD68+ CD206- M1 macrophages | 395.8 (380-490) | 401.3 (118.5-785.3) | 0.603 |
| CD68+ CD206+ M2 macrophages | 2.3 (2.1-6.7) | 0.8 (0.5-2.3) | 0.153 |
| CD11c+ MHC class II+ dendritic cells | 0 (0-0.4) | 0 (0-0.6) | 0.763 |
| PD-L1+ cells | 802.6 (418.6-1206.9) | 683.9 (273.8-1178.5) | 0.897 |
